# Niches and climate-change refugia in hundreds of species from one of the most arid places on Earth

**DOI:** 10.7717/peerj.7409

**Published:** 2019-09-12

**Authors:** Milen Duarte, Pablo C. Guerrero, Mary T.K. Arroyo, Ramiro O. Bustamante

**Affiliations:** 1 Departamento de Ciencias Ecológicas, Universidad de Chile, Santiago, Chile; 2 Instituto de Ecología y Biodiversidad (IEB), Santiago, Chile; 3 Departamento de Botánica, Facultad de Ciencias Naturales y Oceanográficas, Universidad de Concepción, Concepción, Chile

**Keywords:** Refugia, Expansion area, *Malesherbia*, *Chaetanthera*, *Leucocoryne*, *Nolana*, *Eriosyce*, *Schizanthus*

## Abstract

**Background and Aims:**

Global climate change is a major threat to biodiversity worldwide. Several arid areas might expand in the future, but it is not clear if this change would be positive or negative for arid-adapted lineages. Here, we explore whether climatic niche properties are involved in the configuration of climate refugia and thus in future species trends.

**Methods:**

To estimate putative climate refugia and potential expansion areas, we used maximum entropy models and four climate-change models to generate current and future potential distributions of 142 plant species endemic to the Atacama and mediterranean Chilean ecosystems. We assessed the relationship between the similarity and breadth of thermal and precipitation niches with the size of climate refugia and areas of potential expansions.

**Key Results:**

We found a positive relationship between breadth and similarity for thermal niche with the size of climate refugia, but only niche similarity of the thermal niche was positively related with the size of expansion areas. Although all lineages would reduce their distributions in the future, few species are predicted to be at risk of extinction in their current distribution, and all of them presented potential expansion areas.

**Conclusion:**

Species with a broad niche and niche dissimilarity will have larger refugia, and species with niche dissimilarity will have larger expansion areas. In addition, our prediction for arid lineages shows that these species will be moderately affected by climate change.

## Introduction

Global climate change is one of the main factors impacting terrestrial and marine biodiversity in this century (Barnosky et al., 2011). Among the mechanisms that cause biotic impoverishment are the fragmentation and contraction of the geographical distribution of species, which can lead to increases in the degree of threat to these species (Parmesan, 1996; Bakkenes et al., 2002; Thuiller et al., 2008). The contraction of the distribution of species may be due to climate conditions, insofar as associated changes may not meet species’ climatic niche requirements; therefore, a given species may suffer local and/or global extinction (Walther et al., 2002; Thomas et al., 2004; Jiguet et al., 2010; Wiens, 2016). On the other hand, species that will disperse to and track new climatic conditions can expand their distribution (Alarcón & Cavieres, 2015) and move their distribution limits (Felde, Kapfer & Grytnes, 2012).

The responses of organisms to climate change are limited and depend on the speed and intensity of climate change, as well as on biological variables such as physiological tolerance, morpho-functional traits, and life history (Araújo et al., 2013; Parmesan & Hanley, 2015). Predictions of ongoing climate change suggest that abiotic variables will be substantially altered in large geographic areas in a short time period, and species may not be able to adapt to these new conditions (Quintero & Wiens, 2013b; Wiens, 2016). Moreover, the dispersal potential of many species is limited, particularly in low-mobility groups such as terrestrial plants (Higgins & Richardson, 1999; Dullinger, Dirnböck & Grabherr, 2004; Dullinger et al., 2015), and few studies have shown models of dispersion that favor species distribution (Alarcón & Cavieres, 2015). Besides, dispersal of propagules is heavily constrained by habitat destruction and human-induced changes in land use (Hermy & Verheyen, 2007), which act as steadfast barriers to the movement of propagules between unconnected biotopes.

The climatic niche of species can be characterized by evaluating properties such as niche breadth and similarity (Colwell & Futuyma, 1971; Wiens et al., 2009). Climatic niche breadth is the climatic amplitude where one species can exist (Thuiller et al., 2005b). It is possible to distinguish the degree of similarity between these climatic conditions across different localities of the same species, evaluating how similar or different these localities are (Quintero & Wiens, 2013a). Additionally, this niche dimension can be projected onto geographic space (e.g., biotope), thus identifying all suitable areas where species can persist (Soberón & Peterson, 2005; Peterson, 2006; Colwell & Rangel, 2009). This spatial projection has been used to predict changes in the distribution of species predicted by current global climate change (Parmesan & Yohe, 2003; Pearson & Dawson, 2003; Thomas et al., 2004; Thuiller, Lavorel & Araújo, 2005a; Tingley et al., 2009). Thus, changes in the spatial distribution of biotopes enable the identification of the geographical areas that maintain climatic conditions that are suitable for various species after climate change and/or their geographical areas may expand. Furthermore, a comparison of current and future predicted distributions (expected by climate change) can allow for the identification of stable zones that can act as refuge areas for the species (Barnosky, 2008; Trivedi et al., 2008; Williams et al., 2008; Ashcroft, 2010; Keppel et al., 2012; Alamgir, Mukul & Turton, 2015; Serra-Diaz et al., 2015; Stralberg et al., 2015). The size distribution of refugia are relevant for species conservation, since species with small refugia face a greater probability of extinction (Thuiller, Lavorel & Araújo, 2005a).

In the southwestern Andes, it is expected that climate change will modify rainfall regimes, which will increase in summer and decrease in winter (Vera et al., 2006; Sánchez et al., 2015). In recent decades, the western side of the Andes in the Southern Cone has seen cooling in some coastal areas coupled with an increase in temperature at high altitudes (Falvey & Garreaud, 2009). This tendency is likely to continue according to predictions made by the Intergovernmental Panel on Climate Change (IPCC, 2013); by the year 2080, on average, temperatures may rise by 3 °C, and annual rainfall may decrease by 6% in the Atacama Desert, an area that is one of the driest places on Earth (Guerrero et al., 2013). The impact of climate changes will be greater in the mediterranean area of Chile, where a decrease of 50% in annual rainfall and an increase of 2.5 °C in temperature are expected; in contrast, the annual precipitation and temperature in temperate forests of southern South America may increase by 5% and 1 °C, respectively (Christensen et al., 2007).

In this study, we characterized the climatic niche, the size of refugia areas, and the potential expansion size area for 142 plant species from arid western South America. The core task of our study was to evaluate the relationship between niche breadth and similarity of arid-adapted plant species with the size of climatic refugia and potential expansion areas.

## Materials and Methods

### Dataset and study region

This study was conducted in western South America between 25° and 47° latitude, on 142 species from six plant genera: *Chaetanthera* (Asteraceae), *Eriosyce* (Cactaceae), *Malesherbia* (Passifloraceae), *Schizanthus* and *Nolana* (Solanaceae), and *Leucocoryne* (Alliaceae). These genera have received substantial attention by botanists (Gengler-Nowak, 2003; Meudt & Simpson, 2006; Pérez, Arroyo & Medel, 2007; Dillon et al., 2009; Davies, 2010; Guerrero et al., 2011; Guerrero, Durán & Walter, 2011; Jara-Arancio et al., 2014), meaning that good occurrence data are available. To characterize climatic niches and to estimate species distributions, we used the occurrence data obtained directly from a Chilean herbaria (CONC, Herbarium University of Concepción and SGO, National Museum of Natural History), the literature (Guerrero et al., 2013; Jara-Arancio et al., 2014), field trips, and other databases (i.e., the PhD thesis of Meudt, 2004). Bioclimatic variables were obtained from Worldclim (Hijmans et al., 2005). To select variables, we performed a Pearson correlation analysis in ENMTools (Warren, Glor & Turelli, 2010), discarding those variables correlated by over 0.9. A total of 10 variables were retained: mean diurnal range, isothermality, temperature seasonality, maximum temperature of the warmest month, minimum temperature of the coldest month, precipitation seasonality, precipitation of the wettest quarter, precipitation of the driest quarter, precipitation of the warmest quarter, and precipitation of the coldest quarter. The resolution of all climatic layers was 1 km^2^. Managing climatic layers was performed with ArcGIS v. 10.0 (Esri, Redlands, CA, USA).

The future climatic variables were obtained from the National Science Foundation and its project Community Climate System Model, with the CCSMS4.0 model. We used the four representative concentration pathways (Moss et al., 2010), named after a predictable range of radiative values in the year 2100 relative to pre-industrial values in 1750: ~2.6, ~4.5, ~6.0 and >8.5 W m^−2^. These are the most recent global model climate projections that are used in the Fifth Assessment IPCC report (Pachauri et al., 2014).

### Inferring climate refugia

We constructed species distribution models (SDMs) using climate data for current and future 2080 climate conditions. For the future climate condition, we used four scenarios: 2.6, 4.5, 6.0, and 8.5 (in order of increasing gas concentration). SDMs were constructed using MAXENT (Phillips, Anderson & Schapire, 2006; Merow, Smith & Silander, 2013). We correlated occurrence points with 10 climatic variables and used 75% of data for training purposes and 25% model performance. We obtained the model from 10 replicates and a cross-validation procedure for replicate adjustment. Both training and test models obtained area under the curve (AUC) values. For SDM regularization, we selected the average model.

We overlapped the current distribution model of each species with the future projection according to models 2.6, 4.5, 6.0, and 8.5, obtaining four refugia models for each species. All spatial analyses were developed using ArcGIS 10.1 and SDM Toolbox 1.c.1. Finally, we obtained four possible results: range expansion areas (projected areas that are not currently occupied by the species); refugia areas (areas where the current distribution coincides with the future projection); and areas of contraction (current distribution areas, but which are not occupied in the projection figure).

### Climatic niche characterization

To assess niche properties, we selected variables that account for the minimum and maximum ranges of the precipitation and temperature (i.e., the minimum temperature of the coldest month, maximum temperature of the wettest month, precipitation of the wettest quarter, and precipitation of the driest quarter) to describe niche breadth as niche tolerance (Quintero & Wiens, 2013a); further, niche similarity can be described as the variance in niche position of the breadth of the species in all localities.

The climatic niche breadth and similarity of species were assessed with the raw climate values extracted directly from WorldClim using occurrence data (Hijmans et al., 2005). Since it is possible to assess the niche characteristic directly, following Quintero & Wiens (2013a), we calculated the temperature niche breadth as the difference between the minimum temperature of the coldest month and the maximum temperature of the wettest month, as well as the precipitation niche breadth as the difference between the precipitation of the wettest quarter and the precipitation of the driest quarter. Then, the temperature and precipitation within each locality niche breadth were calculated as the differences between their maximum and minimum values; we also calculated their variances and the variance in the position of each locality on the niche axis for all localities in each species as the niche similarity between localities. The raw data can be found in Appendix 1.

Finally, we related the climatic niche results with the size of the refugia and expansion area of each species using generalized lineal models (GLM). For this, we used niche breadth variance (niche breadth) and niche position variance (niche similarity). Refugia and expansion area were the dependent variables, and niche similarity and breadth were the predictor variables.

## Results

**Figure 1:**
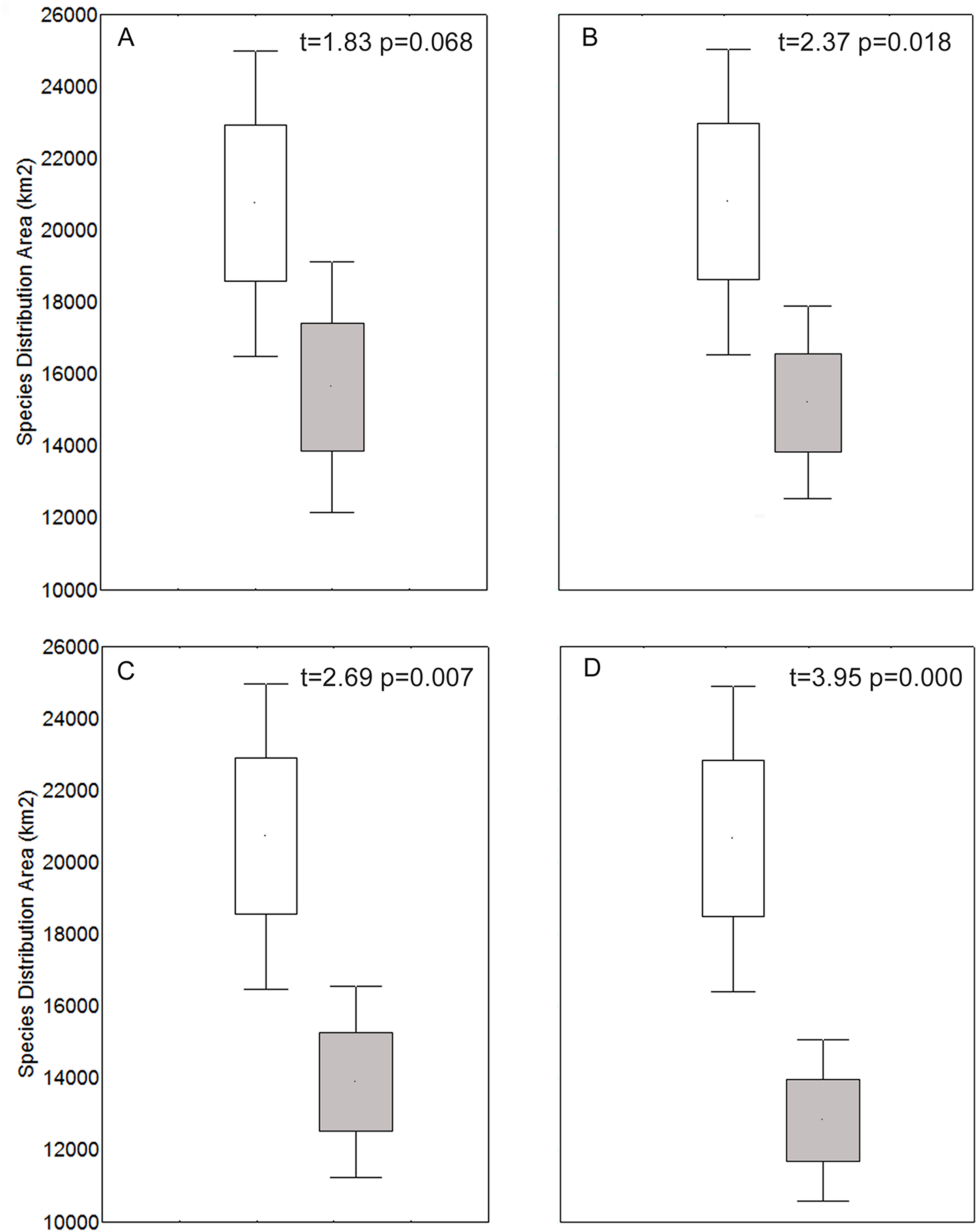
Species distribution area for each current (white) and refugia model (gray), for 2.6 (A), 4.5 (B), 6.0 (C), and 8.5 (D) models. Significant differences are shown between the current and refugia model for models 4.5, 6.0, and 8.5 (*t*-test; *p* < 0.05).

We characterized the niche of the species (Appendix 2). For temperature axes, we found species currently occupying from −12.2 °C to 31.4 °C; for annual precipitation, we found species occupying from 0 mm to 1,104 mm. The spatial analyses indicated that current and future (refugia) species distribution areas were significantly different (*t*-test) for the 4.5, 6.0 and 8.5 models (Fig. 1; *p* < 0.05).

**Figure 2:**
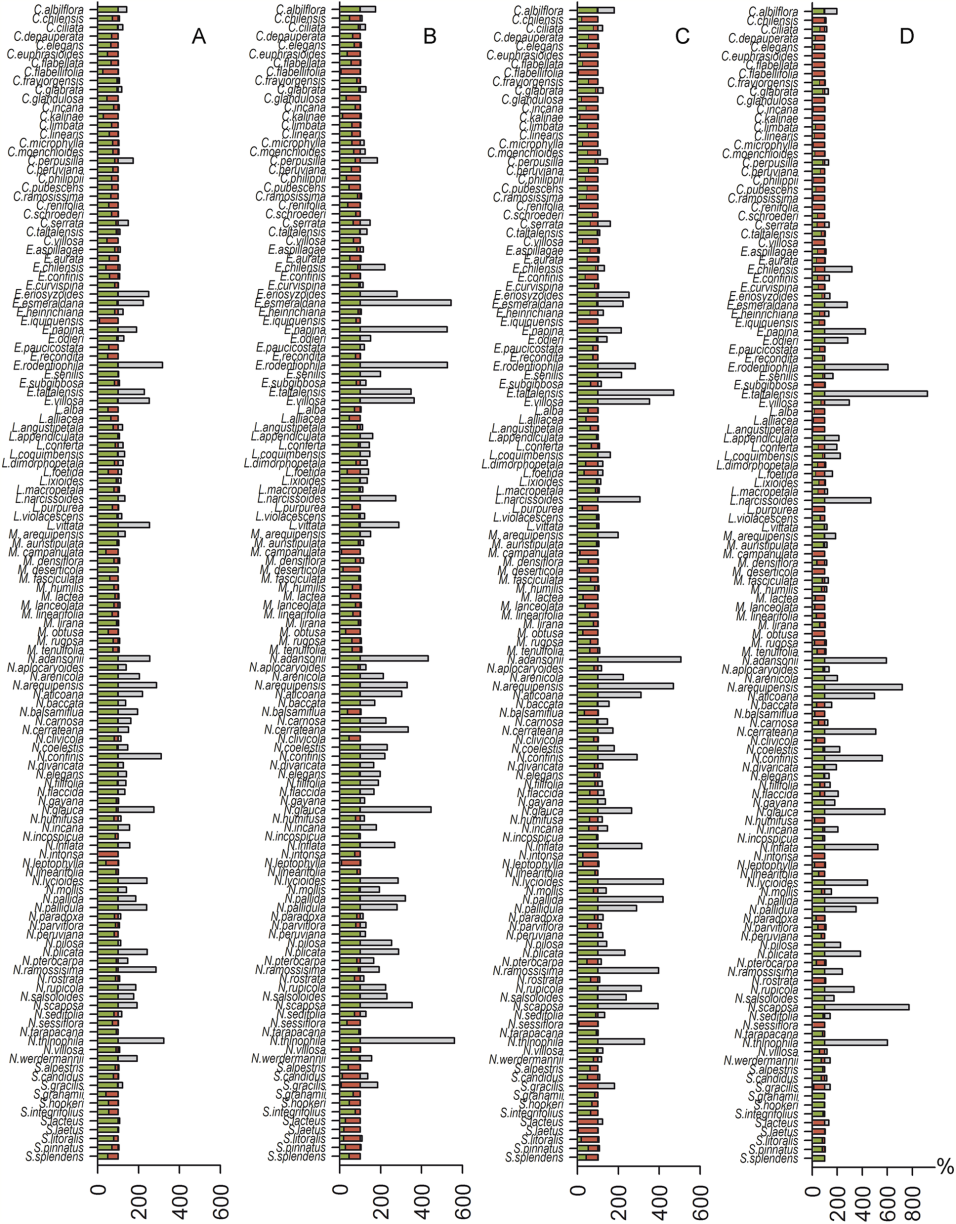
Histogram of contraction area (red), refugia (green) and future expansion (gray) for each species. (A) 2.6 greenhouse scenario model, (B) 4.5 model, (C) 6.0 model, and (D) 8.5 model.

Spatial analyses indicated that the suitable habitat area for species distribution demonstrated reductions of 0%–99.8% for the 2.6 model, 0%–93.9% for the 4.5 model, 0%–100% for the 6.0 model, and 0%–100% for the 8.5 model. We found a potential expansion area of 0%–224.7% for the 2.6 model, 0%–461.1% for the 4.5 model, 0%–406.8% for the 6.0 model and 0%–828.8% for the 8.5 model. The 2.6 model showed one possible extinction (*N. intonsa*) and another species refugia between 0.02%–100%. The 4.5 model showed a 6.10%–100% refugia area; the 6.0 model showed one possible extinction (*E. iquiquensis*) and 0%–100% refugia area; and the 8.5 model showed one possible extinction (*L. purpurea*) and 0%–100% refugia area. Therefore, the least conservative scenario for the three measures used (contraction, refugia, and distribution expansion) is the 8.5 model (Fig. 2), which shows high percentages of contraction (up to 100%), narrow climatic refugia (from 0%), and high expansion values (up to 828.8% of the current distribution).

Significant relationships between temperature niche similarity and the size of refugia were detected for all SDMs (Table 1). The GLM analysis indicated a positive relationship between temperature niche similarity and the four emission scenarios (2.6, 4.5, 6.0, and 8.5), and a positive effect between niche breadth in temperature and the two emission scenarios (6.0 and 8.5). Moreover, we detected positive relationships between niche similarity in temperature for all models and expansion areas (Table 1).

**Table 1:** Relationship between niche breadth and refugia size and size expansion to the four greenhouse scenarios (2.6, 4.5, 6.0, and 8.5). Gray values represent significant results; *p* < 0.05.

	Tests of significance for size refugia (GLM, III)							
	2.6		4.5		6.0		8.5	
	F	*p*	F	*p*	F	*p*	F	*p*
Temperature								
Breadth	0.006	0.940	2.320	0.130	4.581	0.034	17.298	0.000
Position	11.613	0.001	18.594	0.000	17.740	0.000	25.509	0.000
Precipitation								
Breadth	0.040	0.842	0.023	0.881	0.268	0.605	0.849	0.359
Position	0.012	0.913	0.006	0.939	0.032	0.859	0.170	0.681

	Tests of significance for size expansion (GLM, III)							
	2.6		4.5		6.0		8.5	
	F	*p*	F	*p*	F	*p*	F	*p*
Temperature								
Breadth	1.589	0.210	1.581	0.211	1.682	0.197	1.274	0.261
Position	17.402	0.000	16.139	0.000	16.591	0.000	14.243	0.000
Precipitation								
Breadth	0.376	0.541	0.412	0.522	0.342	0.560	0.111	0.740
Position	0.061	0.805	0.083	0.774	0.062	0.804	0.111	0.739

## Discussion

Niche breadth is positively correlated with the geographic distribution of species (Slatyer, Hirst & Sexton, 2013) and the survival of species (Saupe et al., 2015). In terms of climate change, we found that climatic niche breadth and similarity are positively correlated with refugia size and temperature, a result consistent with previous evidence (Thuiller, Lavorel & Araújo, 2005a). In our arid lineages, species with wide climatic niches and more dissimilar niches (non-grouping niche position) would be less affected by global warming compared to species with more narrow climatic niches. In addition, species with narrow distributions or habitat specialist species may be more prone to extinction after climate change (Johnson, 1998), since there is a positive relationship between the size of the distribution area and species abundance (Brown, 1984; Gaston, 1996).

We believe that for species with narrow climatic niches, the presence of climate refugia can be subject to the magnitude of climate change in their geographic area. Alarcón & Cavieres (2018) found a positive relationship between niche breadth and change in species’ distribution with increasing elevation; however, as the latitude increases, the relationship is reversed, and species with wide niches present greater changes in their distribution, which reaffirms the idea that the niche breadth–refugia relationship could be modified by the magnitude of change in a specific geographical area. It is important to incorporate other factors, such as latitude or gradients in future studies.

As a consequence of the fact that species hold limited potential to adapt to the warmer and drier climatic conditions, together with dispersion constraints, the number of species threatened by global warming should increase in the future (Thomas et al., 2004; Thuiller et al., 2005b, 2006; Keppel et al., 2012). Although our study in arid lineages showed significant differences between the current distribution and potential future areas (refugia) only some species present a high risk of extinction under expected future climate-change scenarios: for the most conservative scenario (2.6), 63% of the evaluated species will retain over 80% of their distribution, 19% will retain between 60% and 80%, 14% will retain between 40% and 60%, 3% will retain between 20% and 40%, and only 1% will retain less than 20% of its distribution. For the IUCN (2012), a species is considered endangered if its population has reduced by at least 50% in 10 years or has a extend of occurrence less than 5,000 km^2^, or has an area of occupancy less than of 500 km^2^; hence, our results suggest that the species studied should not be at high risk. Although there are few studies in SDMs for semi-arid and arid ecosystems, our results are similar to those found in Namibia, for example. In that study, it was predicted that less than 5% of the species could experience a complete range reduction by 2080, although it is expected that more than 47% will have a range reduction of at least 30% by the year 2080 (Thuiller et al., 2006). However, in our study, species that showed a greater than 50% reduction in their distribution were species of importance due to their high extinction risk. *E. chilensis* and *E. recondita* are considered endangered in their endemic distribution.

From an evolutionary perspective, a study based on the effects of climate change on endemic species in Sahara-Sahel showed that some groups with a high capacity to adapt to global change (for example, those with a high dispersal capacities) may be able to colonize distinct areas, while groups with low adaptive capacity may be more vulnerable to extinction (Vale & Brito, 2015). This result is consistent with the finding from study on arid-adapted plants, where desert plants may be resilient to climate change since they presented with positive population growth rates (Salguero-Gómez et al., 2012). Also, at an intraspecific level, semiarid plants could present a better response to climate change in more arid populations because of the greater phenotypic plasticity of these populations in comparison with more mesic populations (Lázaro-Nogal et al., 2015). Persistence against climate change may be favored by species with seed dormancy, since seeds may resist long periods of drought (Clauss & Venable, 2000), while rapid life cycles and fast reproductive processes allow species to take advantage of short windows of ecological opportunities when resources are abundant, such as episodic rainfalls (Aronson et al., 1993), which allow water to be retained in water-scarce conditions. However, there is evidence of an evolutionary lag time for *Chaetanthera*, *Malesherbia* and *Nolana* to adapt to new, more severe arid conditions, and thus rapid adaptation to ongoing climate change may be unlikely (Guerrero et al., 2013). This tells us that the study of arid lineages and their future in the face of climate change is still in process. Therefore, describing these evolutionary advantages is also important when evaluating the future distributions of various species. For example, in our study, some species of the genus *Leucocoryne* (geophite) showed a large niche breadth and low similarity; at the same time, it is known that these plants have bulbous structures that enable it to store water in prolonged droughts (Jara-Arancio et al., 2014).

By detecting areas of possible expansion for species distribution, it is possible to propose areas that could potentially benefit from conservation efforts and ecological restoration, as reforestation with those species that present expansion in those areas (Padonou et al., 2015) and human-assisted introductions to maximize the native forests’ connectivity (Hannah et al., 2008), could attenuate the impact generated by the contraction of natural species distribution. This conservation method is currently being incorporated for conservation planning and ecological restoration (Yang et al., 2013; Ardestani et al., 2015; Remya, Ramachandran & Jayakumar, 2015). In our case, this could be very useful for arid lineages, due to the intensification of mining activity in the western Andes (Cisternas & Gálvez, 2014), and this study could serve as the impetus for initiating restoration in that area. Proposing areas of expansion for these arid lineages would counteract the effect of global change and, in turn, fill gaps in conservation, as in the case of groups such as cacti, which are poorly conserved in non-take areas (Duarte et al., 2014). Therefore, areas of both refugia and expansion could be subject to concrete conservation efforts, and they may also be used in environmental policies and conservation planning.

## Conclusions

This work provides new knowledge on which properties could define the future distribution of species throughout the course of global climate change. We have found that species with a broad niche and niche similarity will have a larger refuge. In addition, those species with niche similarity will have larger expansion areas than species with low similarity. The species evaluated belong to semi-arid ecosystems, which seldom been evaluated in relation to their future distribution. Our prediction in arid lineages shows that these species will be moderately affected by climate change. For this reason, we suggest taking conservative measures to protect these lineages in the places where they are currently distributed, which will serve as future areas of refuge in the face of climate change.

## Supplemental Information

10.7717/peerj.7409/supp-1 Supplemental Information 1 Raw data for the construction of climatic niches of the species. Each folder represents the data associated with a plant genera, and contains two files: B16_b17 and B5_b6, which describe the climatic variables associated with each location of each species. The former contain the values related to precipitation, while the latter contain the values associated with the temperatures.

10.7717/peerj.7409/supp-2 Supplemental Information 2 Niche breadth and position over all localities for each species. Graph, WLS Ratio (WLS-Ratio as the average of proportions WL-NB / SNB; WL-NB as temperature and precipitation within each Locality Niche Breadth and SNB the overall Temperature and Precipitation Species Niche Breadth), NBV (as the variance of temperature and precipitation WL-NB) and NPV (as the variance in the position of each locality on the niche axis for all localities in each species).

## Additional Information and Declarations

### Data Availability

The following information was supplied regarding data availability: The raw data are available in the Supplemental File.
