# Supplementary material for: Niches and climate-change refugia in hundreds of species from one of the most arid places on Earth
Source: PeerJ. 2019 Sep 12;7:e7409. doi: 10.7717/peerj.7409 (PMC6745186; doi:10.7717/peerj.7409)
Supplement: Supplemental Information 2 — Graph, WLS Ratio (WLS-Ratio as the average of proportions WL-NB / SNB; WL-NB as temperature and precipitation within each Locality Niche Breadth and SNB the overall Temperature and Precipitation Species Niche Breadth), NBV (as the variance of temperature and precipitation WL-NB) and NPV (as the variance in the position of each locality on the niche axis for all localities in each species). [file peerj-07-7409-s002.doc]

1. Niche breadth over all localities for each species and genus. Graph, WLS Ratio (WLS-Ratio as the average of proportions WL-NB / SNB; WL-NB as temperature and precipitation within each Locality Niche Breadth and SNB the overall Temperature and Precipitation Species Niche Breadth), NBV (as the variance of temperature and precipitation WL-NB)and NPV(as the variance in the position of each locality on the niche axis for all localities in each species).

a)Genus *Chaetanthera* (Asteraceae)

| *Precipitation* | 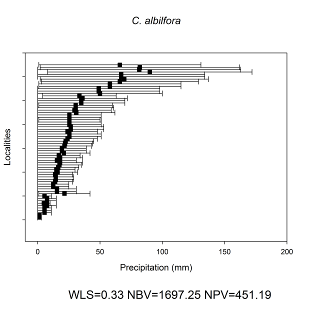 | 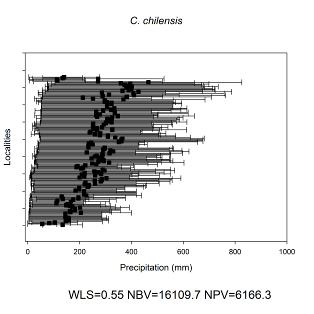 | 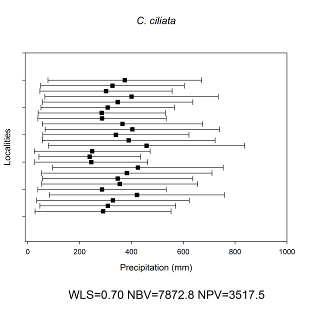 |
| --- | --- | --- | --- |
| 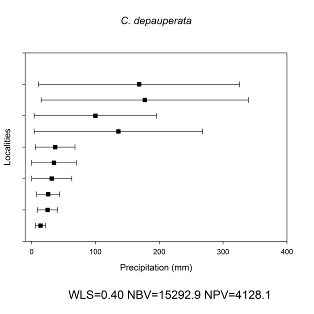 | 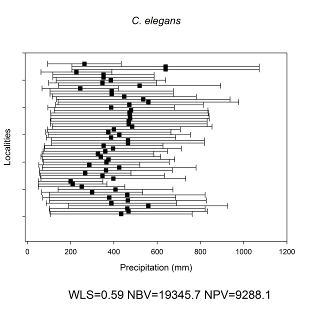 | 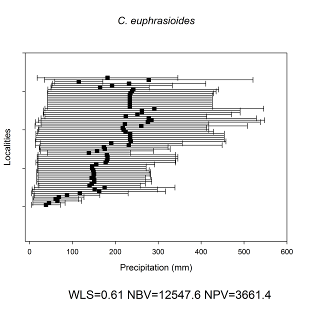 | 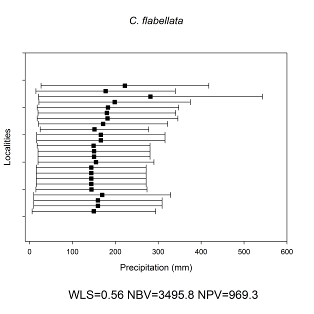 |
| 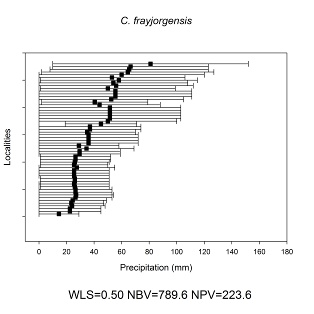 | 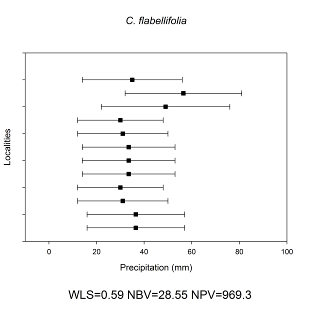 | 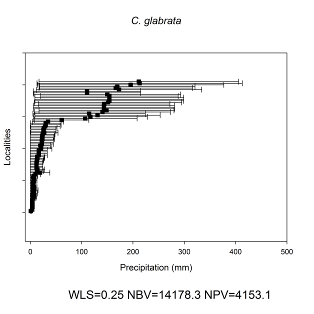 | 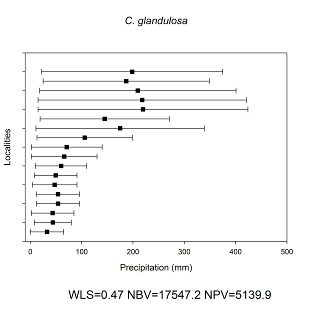 |
| 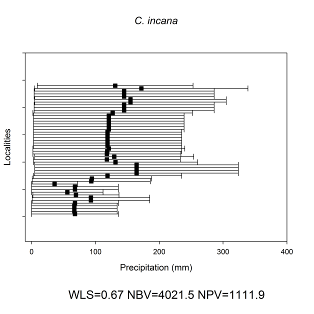 | 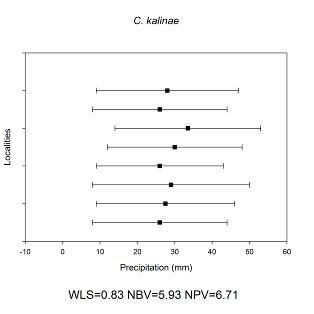 | 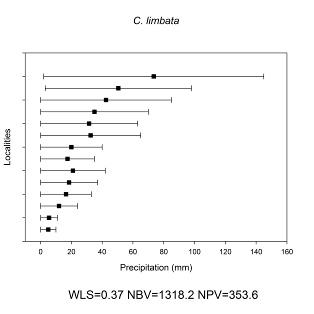 | 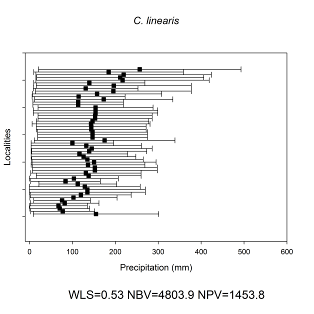 |
| 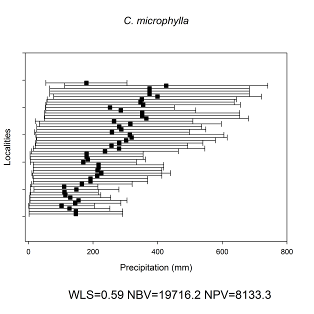 | 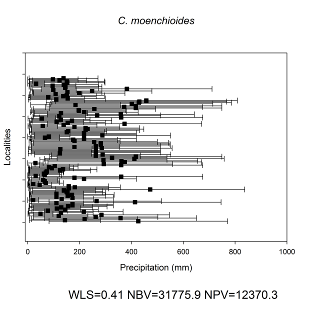 | 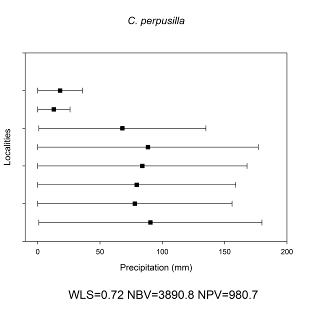 | 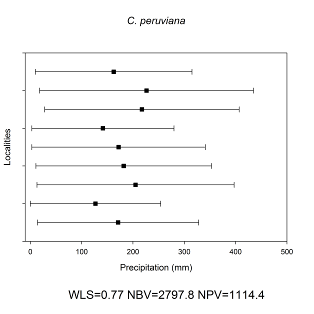 |
| 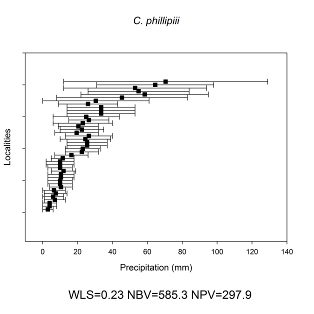 | 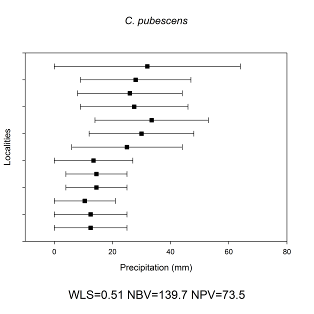 | 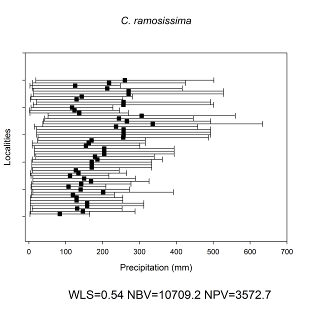 | 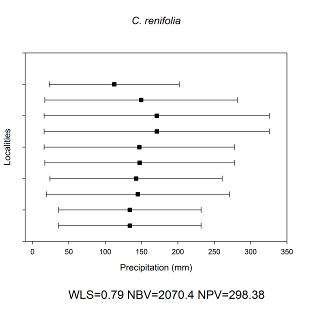 |
| 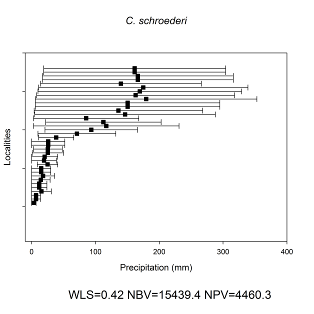 | 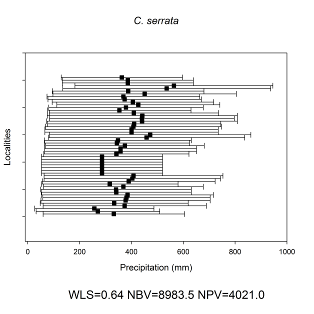 | 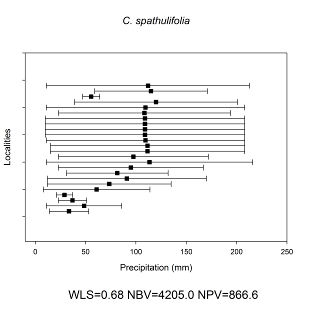 | 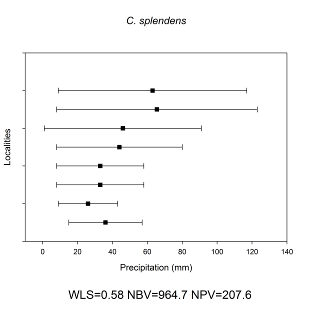 |
| 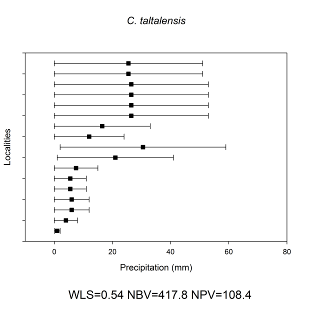 | 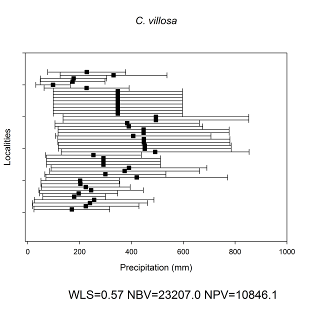 |  |  |

| *Temperature* | 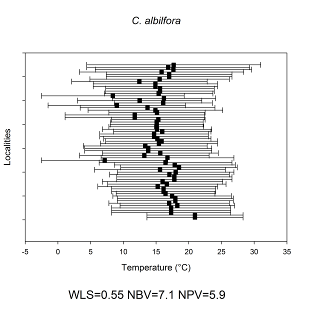 | 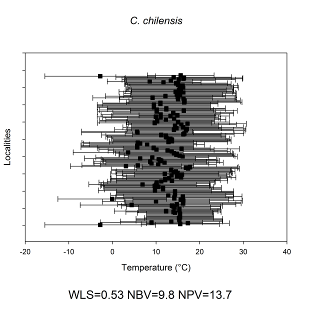 | 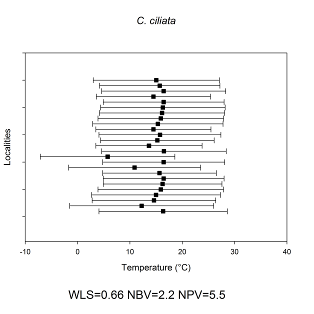 |
| --- | --- | --- | --- |
| 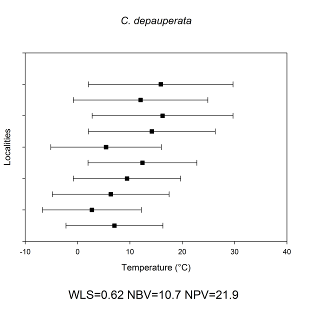 | 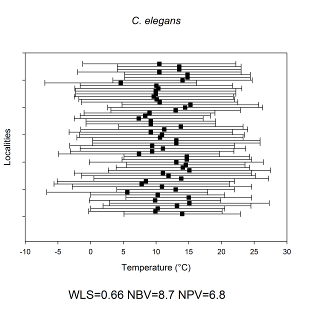 | 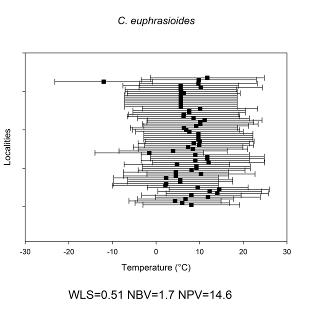 | 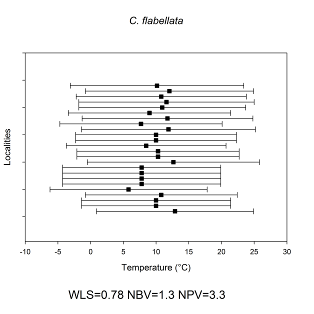 |
| 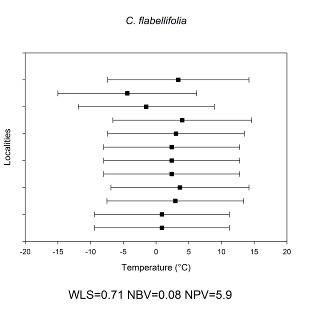 | 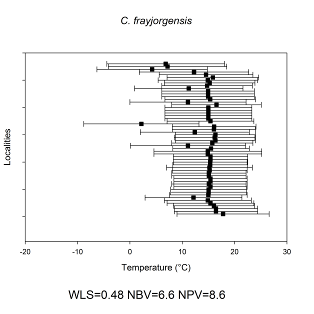 | 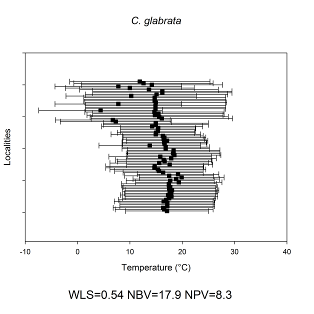 | 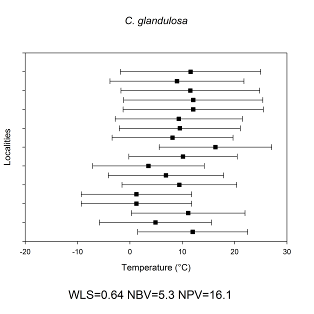 |
| 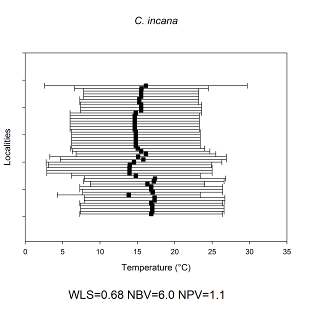 | 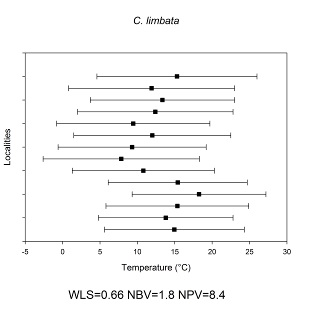 | 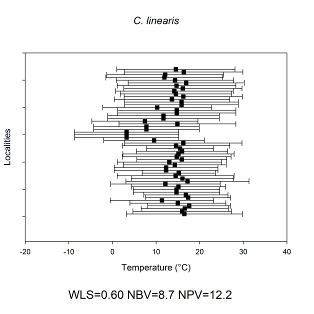 | 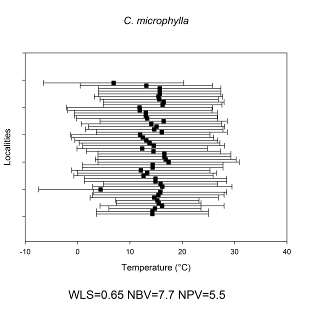 |
| 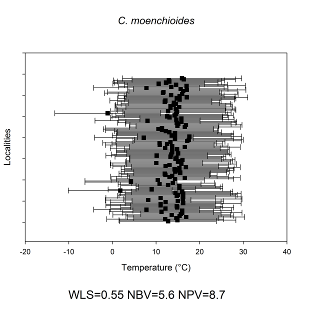 | 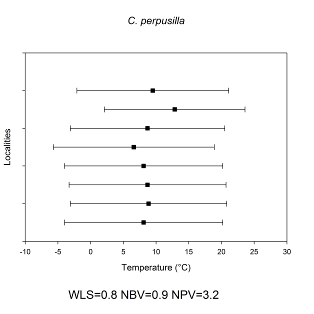 | 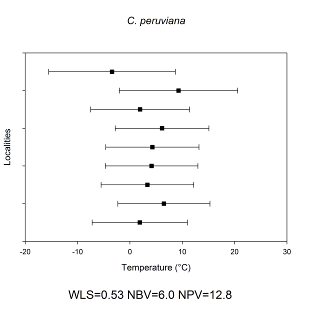 | 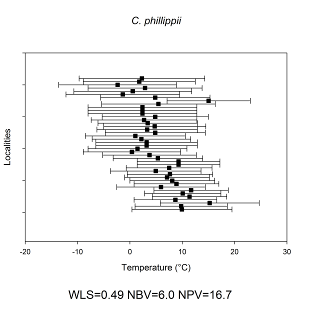 |
| 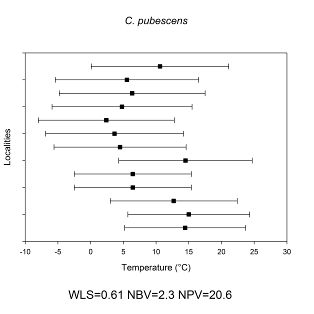 | 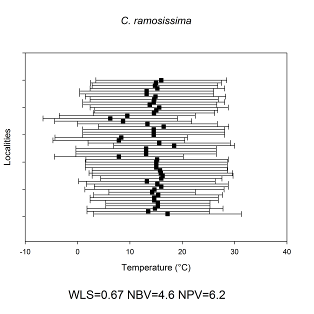 | 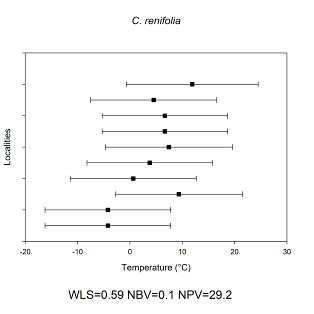 | 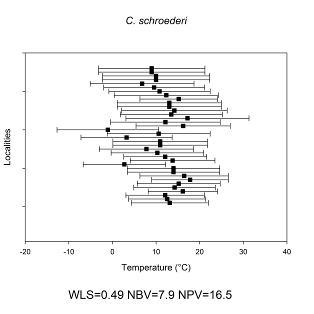 |
| 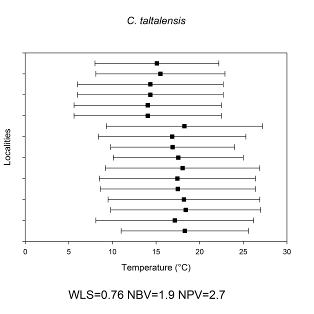 | 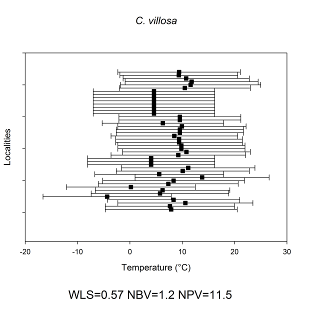 |  |  |

b) Genus *Eriosyce* (Cactaceae)

| *Precipitation* | 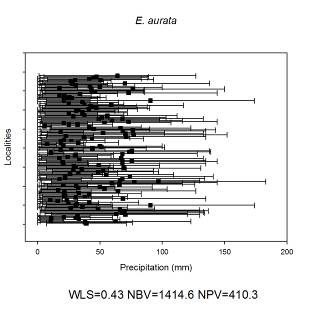 | 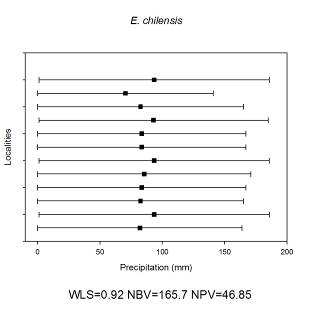 | 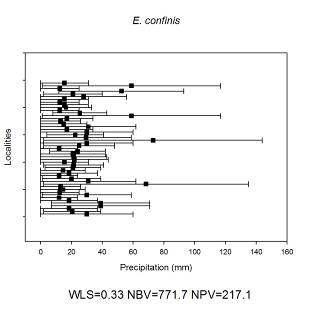 |
| --- | --- | --- | --- |
| 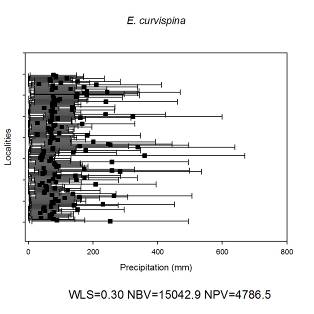 | 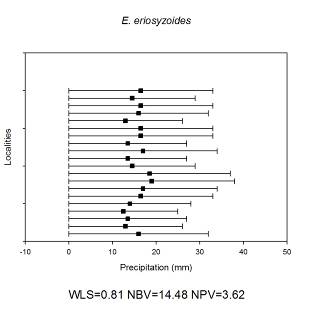 | 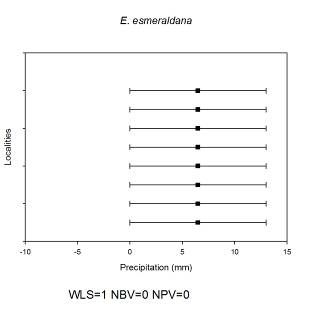 | 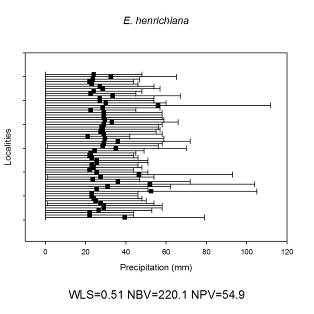 |
| 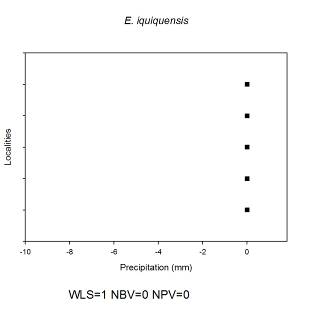 | 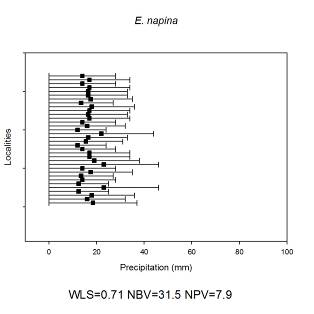 | 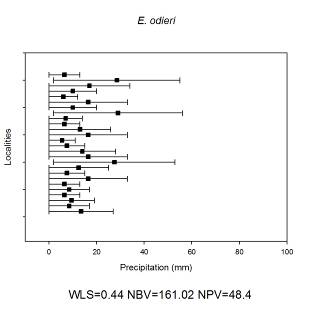 | 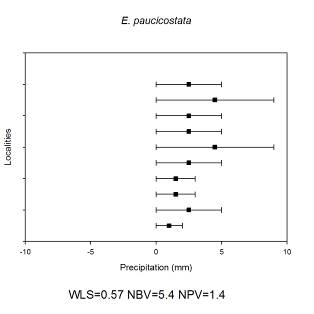 |
| 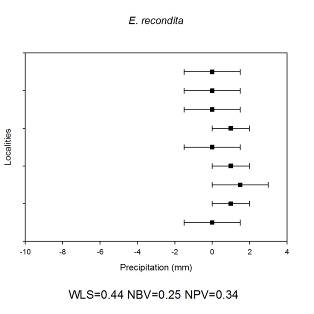 | 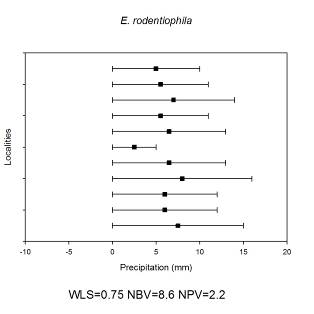 | 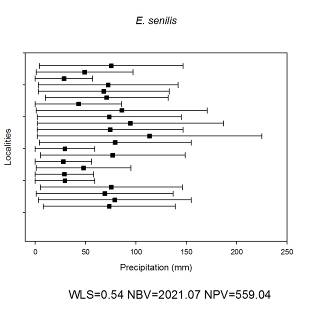 | 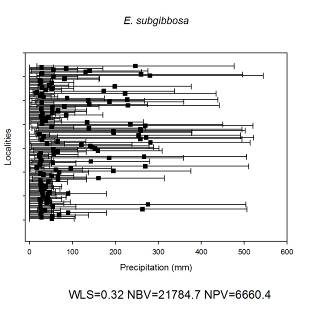 |
| 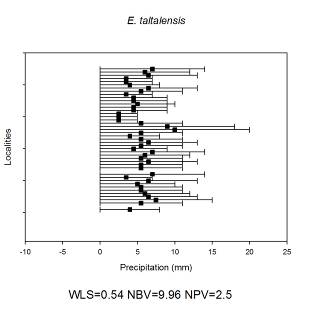 | 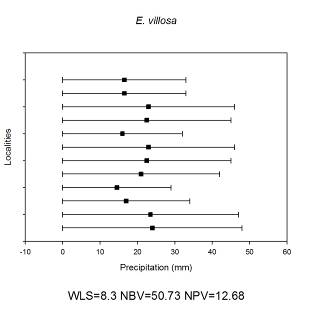 | 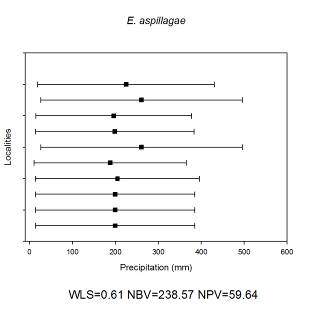 |  |

| *Temperature* | 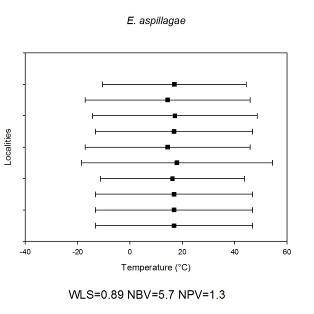 | 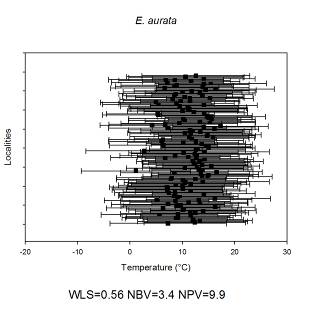 | 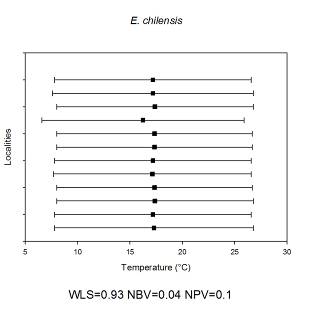 |
| --- | --- | --- | --- |
| 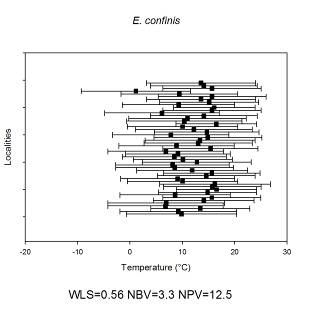 | 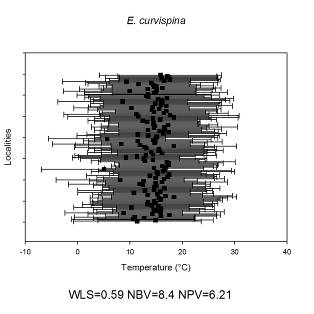 | 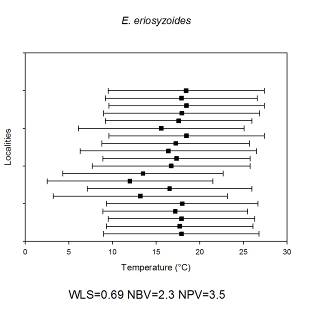 | 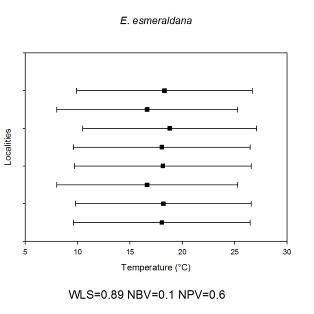 |
| 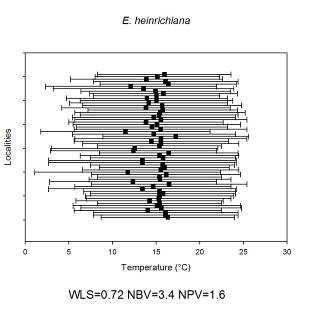 | 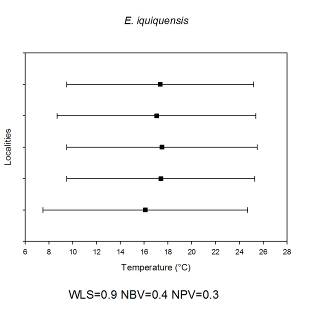 | 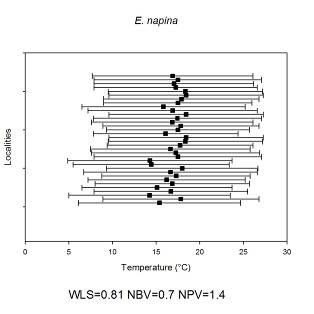 | 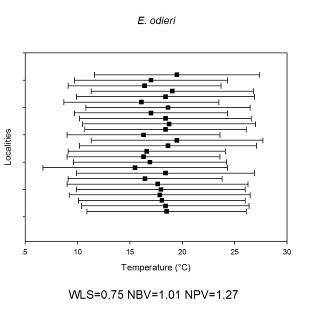 |
| 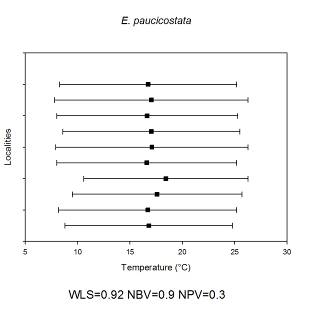 | 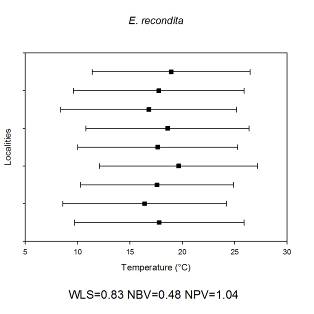 | 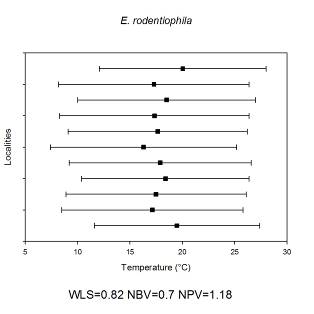 | 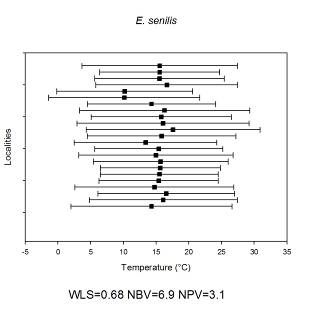 |
| 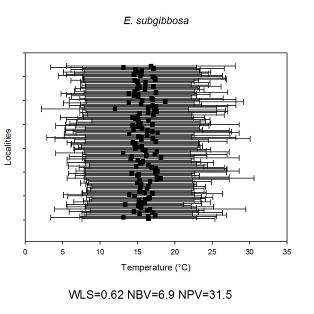 | 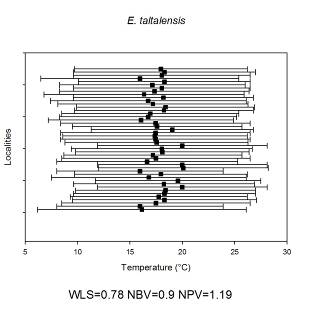 | 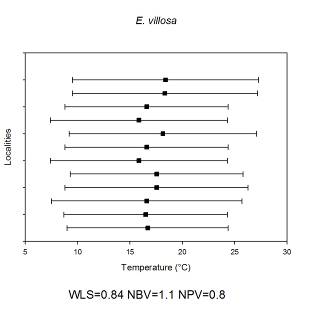 |  |

d) Genus *Leucocoryne* (Alliaceae).

| *Precipitation* | 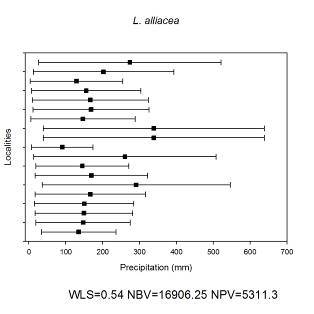 | 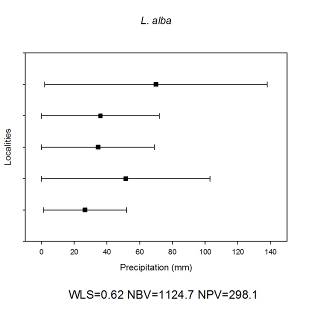 | 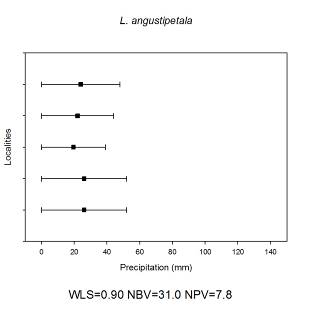 |
| --- | --- | --- | --- |
| 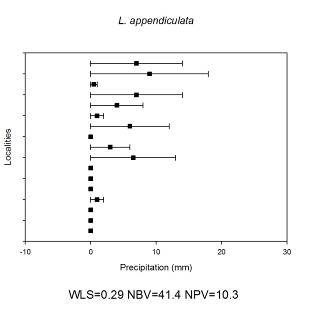 | 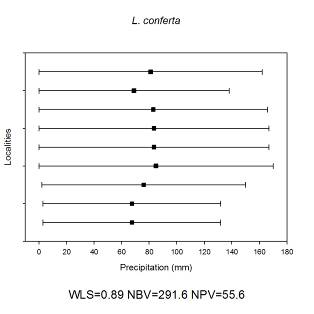 | 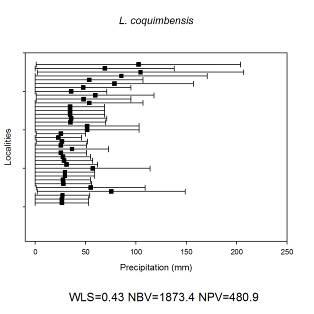 | 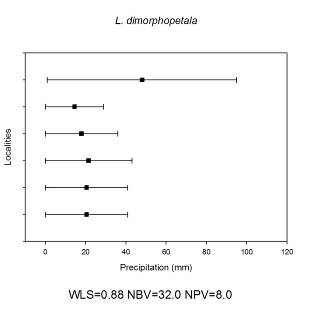 |
| 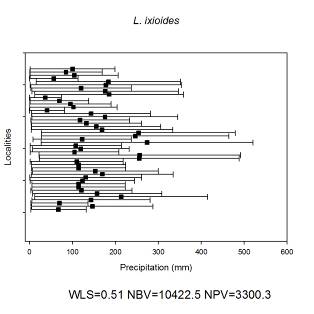 | 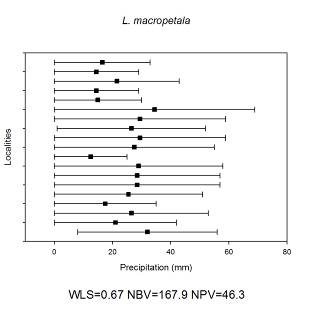 | 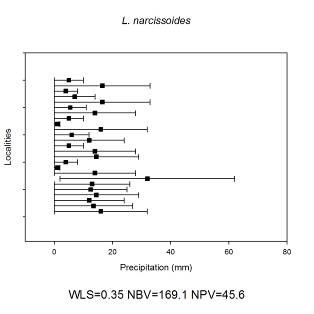 |  |
|  |  |  |  |

| *Temperature* |  |  |  |
| --- | --- | --- | --- |
|  |  |  |  |
|  |  |  |  |
|  |  |  |  |

e) Genus *Malesherbia* (Passifloraceae).

| *Precipitation* |  |  |  |
| --- | --- | --- | --- |
|  |  |  |  |
|  |  |  |  |
|  |  |  |  |

| *Temperature* |  |  |  |
| --- | --- | --- | --- |
|  |  |  |  |
|  |  |  |  |
|  |  |  |  |

f) Genus *Nolana* (Solanaceae).

| *Precipitation* |  |  |  |
| --- | --- | --- | --- |
|  |  |  |  |
|  |  |  |  |
|  |  |  |  |
|  |  |  |  |
|  |  |  |  |
|  |  |  |  |
|  |  |  |  |
|  |  |  |  |
|  |  |  |  |
|  |  |  |  |
|  |  |  |  |

| *Temperature* |  |  |  |
| --- | --- | --- | --- |
|  |  |  |  |
|  |  |  |  |
|  |  |  |  |
|  |  |  |  |
|  |  |  |  |
|  |  |  |  |
|  |  |  |  |
|  |  |  |  |
|  |  |  |  |
|  |  |  |  |
|  |  |  |  |

f) Genus *Schizanthus* (Solanaceae).

| *Precipitation* |  |  |  |
| --- | --- | --- | --- |
|  |  |  |  |
|  |  |  |  |

| *Temperature* |  |  |  |
| --- | --- | --- | --- |
|  |  |  |  |
|  |  |  |  |
